# Quality of Life: The Interplay between Human Behaviour, Technology and the Environment

**DOI:** 10.3390/ijerph16245106

**Published:** 2019-12-13

**Authors:** Joost van Hoof, Deirdre M. Beneken genaamd Kolmer, Erwin de Vlugt, Sanne I. de Vries

**Affiliations:** 1 Faculty of Social Work & Education, The Hague University of Applied Sciences, Johanna Westerdijkplein 75, 2521 EN Den Haag, The Netherlands; 2 Department of Spatial Economy, Faculty of Environmental Engineering and Geodesy, Wrocław University of Environmental and Life Sciences, ul. Grunwaldzka 55, 50-357 Wrocław, Poland; 3 Faculty of Health, Nutrition & Sports, The Hague University of Applied Sciences, Johanna Westerdijkplein 75, 2521 EN Den Haag, The Netherlands; d.m.beneken@hhs.nl (D.M.B.g.K.);; 4 Faculty of Technology, Innovation & Society, The Hague University of Applied Sciences, Johanna Westerdijkplein 75, 2521 EN Den Haag, The Netherlands; e.devlugt@hhs.nl

Quality of life is an umbrella term for the quality of the various domains in life. What does quality of life mean to a person? Quality of life has a different meaning for every individual, as every person has his or her own set of expectations. Contexts in which people live, by one’s values, and one’s personal goals in life [1,2,3,4], as well as resilience in coping with stressful situations steer these expectations. Additionally, to a large extent, quality of life as a concept is subjective and multidimensional. The World Health Organization (WHO) defines quality of life as an individual’s perception of their position in life in the context of the culture and value systems in which they live and in relation to their goals, expectations, standards, and concerns [5]. It is a broad ranging concept that the person’s physical health, psychological state, personal beliefs, social relationships, and their relationship to salient features of their environment affect in a complex way [5]. Quality of life serves a reference against which you can measure the various domains of your own life or that of other individuals, and that can change over time. This definition of the World Health Organization encompasses many elements of daily living, including features of the individual and the environment around us, which can either be the social environment, the built environment, or other environmental aspects. This is one of the rationales for the special issue on “*Quality of Life: The Interplay between Human Behaviour, Technology and the Environment*”. This special issue is a joint project by the Centre of Expertise Health Innovation of the Hague University of Applied Sciences in The Netherlands.

The main focus of this Special Issue is how optimising the interplay between people, the environment, and technology can enhance people’s quality of life. The focus of the contributions in this special issue is on the person or end-user and his or her environment, both the physical, social, and digital environment, and on the interaction between (1) people, (2) health, care, and systems, and (3) technology. Recent advances in technology offer a wide range of solutions that support a healthy lifestyle, good quality of life, and effective and efficient healthcare processes, for a large number of end-users, both patients/clients from minus 9 months until 100+ years of age, as well as practitioners/physicians. The design of new services and products is at the roots of serving the quality of life of people.

In a special issue of the International Journal of Environmental Research and Public Health on “*Quality of Life: The Interplay between Human Behaviour, Technology and the Environment*”, a total of eighteen papers [6,7,8,9,10,11,12,13,14,15,16,17,18,19,20,21,22,23] were recently published on different topics that are related to this subject matter. Of the published papers, four papers [6,7,8,9] were on age-friendly environments, describing the relation of how the environment and technology influences people as they age. A total of seven papers [10,11,12,13,14,15,16] were published regarding how technology can support (older) people in their daily lives in various ways. Three papers [17,18,19] were published on special care environments and their design, and how the design influences the users of such environments. Finally, four papers [20,21,22,23] were published on the design and implementation of assistive technologies, which lead to a better fit between the technology and the end-users.

The first four papers mainly focus on the interplay between older people and the built environment. The paper by van Hoof et al. [6] explores and describes the challenges that are encountered when making cities age-friendly in Europe, including the creation of inclusive neighbourhoods and the implementation of technology for ageing-in-place. Examples from projects in two age-friendly cities in The Netherlands and Poland are shown to illustrate the potential of making cities more tuned to the needs of older people and to identify important challenges for the next couple of years.

The second paper by van Hoof et al. [7] focuses on the quality of the thermal environment in homes of older South Australians. Ageing brings about physiological changes that affect people’s thermal sensitivity and thermoregulation. Modifications to aid thermal comfort are not always considered. While using a qualitative approach, this study aims to understand the thermal qualities of the existing living environment of older South Australians, their strategies for keeping cool in hot weather and keeping warm in cold weather, and to identify existing problems that are related to planning and house design, and the use of heating and cooling.

Rusinovic et al. [8] presented a qualitative study on senior co-housing communities in The Netherlands. Such communities offer an in-between solution for older people who do not want to live in an institutional setting, but prefer the company of their age peers. Their contribution adds to the literature by scrutinizing the benefits and drawbacks of senior co-housing, with special focus on social support and the implications for the experience of loneliness. The research shows that co-housing communities offer social contacts, social control, and instrumental and emotional support.

Technology has become essential for contemporary and future societies over the decades, and even more imperative as the decades move on. The paper by Marston and van Hoof [9] presents a critique of the WHO’s Age-Friendly Cities & Communities model, as technology is not explicitly considered in this model. This paper discusses the gaps in the WHO’s model in the field of technology and provides insights and recommendations regarding the expansion of the model for application in the context of countries with a high human development index (HDI) that wish to be fully age-friendly.

The paper by Petrovčič et al. [10] is the first of a series of seven papers [10,11,12,13,14,15,16] on the use of technology by community-dwelling older people, as well as in long-term care facilities and among younger cohorts. The paper presents the results from a population-based survey from Slovenia on the predictors of seniors’ interest in assistive applications (apps) on smartphones. The Cycle of Technology Acquirement by Independent-Living Seniors (C-TAILS) model was recently proposed for studying the interplay between the acceptance factors for technology by integrating the personal, social, and technological domains of seniors’ daily lives. Awareness that a range of factors play different roles in the acceptance of assistive apps is needed for designing viable interventions for seniors.

Juul et al. [11] focused their research efforts on older people living in residential aged care facilities. Their study investigated the potential for new technologies to enhance the quality of life and facilitate meaningful engagement in physical and social activities among culturally and linguistically diverse residents and staff in care facilities. New technologies can be used to increase meaningful physical and social engagement, including transcending language and cultural barriers. However, the successful application of these new technologies is dependent on their integration into the daily routine and social relationships of staff and residents, with the full support of management.

Svensson [12] aimed to identify the challenges that are associated with collaboration in the daily healthcare work practice, among professionals in different healthcare organisations as well as the patients themselves and their relatives, in relationship with the use of IT systems. The study was based on focus groups with physicians, nurses, an assistant officer, a physical or occupational therapist, and a relative. The challenges identified in the study include insufficient information exchange, inconsistencies in communication, differences in the use of IT systems, and deficient coordination.

Forsman and Svensson [13] described frail older persons’ experiences of hospital care, information, and participation when being an inpatient at a hospital. Patients experienced not receiving information regarding their care and rehabilitation, or receiving such information in noisy surroundings. They experienced situations of misunderstanding that were related to their medication, which indicates the need for the appropriate discharge calls for frail older patients. They expressed feelings of distress concerning the future, being caused by hasty admissions or relatives’ problems to handle the situation. The results highlight the need to receive appropriate information and participate in decision-making when hospitalised.

Ehn et al. [14] investigated seniors’ and health care professionals’ perceptions on the needed contributions and qualities of digital technology-based motivation support for seniors’ physical activity. The main findings are that seniors and health care professionals both believed digital technology should support and make physical activity more enjoyable in ways to strengthen seniors’ control and well-being. However, seniors emphasised support for social interaction, while care professionals also requested support for increasing seniors’ insight into physical activity and for facilitating their dialogue with seniors.

Mannheim et al. [15] provided a paper on digital technology for older people. Ageing is stereotypically framed as a problem and older adults are often considered to be frail and incompetent. Not surprisingly, much of the technologies developed focus on care. The exclusion of older adults from research and design of digital technology is often based on such negative stereotypes. The authors made the case for the inclusion, rather than exclusion, of older adults in the design process and research of digital technology is essential if technology is to fulfill the promise of improving well-being.

Ten Bruggencate et al. [16] studied the role of technology that enables social interaction to fulfil the social needs of older people. Social technology concerns any technology that facilitates social interactions and influences social processes between people, including social networks and smartphones. By analyzing interviews held with older people, who regularly use some form of social technology, the researchers found that technology might strengthen the existing social relationships and social structures. Social technology also brings depth and fun to the social contacts and enriches one’s social life. It also gives a sense of safety and peace of mind. The findings might help in implementing and designing new social technologies.

The next three papers are related to the environment of care. The first of this set of three papers is a study by Monsuez et al. [17] on Le Louvre à l’Hôpital. Arts and cultural programs were reported to enhance the quality of life of hospitalized patients with anxiety and depressive symptoms. The Le Louvre à l’Hôpital study presents a new approach, in which the museum moves to the hospital by interactively displaying and discussing artworks with patients.

The study by Kotradyova et al. [18] deals with wood and its impact on humans and environmental quality in health care facilities. The paper presents the application of natural materials, especially wood, which are relevant for human well-being in the built environments of health, social, and day care facilities. The use of wooden materials verifies their regenerative and positive impact on the human nervous system, through the appealing aesthetics (colour, texture, and structures), high contact comfort, pleasant smell, possibility to regulate air humidity, volatile organic compound emissions, and acoustic well-being in the space. The results show how the application of solid wood contributes to well-being of patients and occupants, and it is suitable for application in the design of health care, as well as social and day care facilities.

The study by Gao et al. [19] deals with the utilisation of a mobile dental vehicle for oral healthcare in rural areas. Oral diseases remain one of the major global public health challenges, and the worldwide urban-rural disparities in oral health are significant. Mobile dental vehicles have been proposed as an alternative strategy for supplementing the traditional oral healthcare in many regions. Their article discusses the use of mobile dental vehicles as a solution to urban-rural inequality in receiving oral healthcare.

The last set of four papers [20,21,22,23] deals with the design and implementation of assistive devices and prosthetics and orthoses. First of all, Holtkamp et al. [20] present a paper regarding non-satisfaction with assistive devices. According to Holtkamp, orthopedic engineers have too little insight in the different areas of life of patients, leading to deficient design requirements. The authors present the Triple I model to understand the different areas of life of patients. The Triple I model is elaborated for assistive devices and it offers an associated methodology to orthopedic engineers to systematically map the different areas of life of patients, to understand the requirements for every area, and to explore the conditions. These insights will lead to more tailored design requirements and potentially more satisfactory assistive devices.

The second paper by Manero et al. [21] deals with the implementation of 3D printing technology in the field of prosthetics. Prosthesis needs for children are particularly complex, due in part to their growth rates. Access to a device can have a significant impact on a child’s psychosocial development. This paper examines new research and development of prostheses by the maker community and nonprofit organizations, as well as a novel case study exploring the development of technology and the training methods that are available for users.

The third paper by Rytterström et al. [22] explored the meanings of parents’ and teachers’ other-oriented hope related to eye gaze technology for children with severe disabilities. The eye gaze-controlled computer creates new imaginations of a brighter future for the child, but it also becomes a source for motivation and action in the present.

The fourth paper by Bong et al. [23] dealt with tangible user interfaces, which enables people to interact with the digital world through everyday physical objects. Such interfaces should offer more intuitive digital environments for older people. The study investigated the relationship between older people’ s technology acceptance and quality of life, the changes in these outcome measures after using a tangible user interface, and the associations between them. The study found some positive changes in technology acceptance after the use of the tangible user interface, which might have implications for technology designers and developers, as well as clinicians and other health professionals.

In this special issue, eighteen papers have been published that focus on the interplay between people, his or her environment, and technology. The interaction between the different ‘players’ in the field—the patients, clients, end-users, designers and formal and informal carers and other (allied) health professionals–and the physical, social, and digital environment is complex. This complexity, which is reflected in the differences in stakeholder needs, has its implications on the design of technology and buildings, as well as the environment around us [24,25,26,27,28,29]. Former research has shown that the relationship between human behaviour and the environment is not only country-, continent-, or culture-specific, but also age-, purpose-, and behaviour specific. For example, the built environment correlates of children’s walking and cycling behaviour differ by commuting mode (walking versus cycling) and purpose (such as recreation and transportation) [30]. The relationship also differs per time-segment of the day [31,32]. Overall, this special issue has added to the knowledge base on the interaction between human behaviour and the environment. Now that technology has become such an integral part of our everyday life, technology can be seen as an extra dimension that has to be taken into account when examining quality of life and the interplay between people and environment [9]. Ideally, the perspectives of all stakeholders should be covered. Individuals differ in their values and motives with regard to health, care, their desired living environment, and technologies [33,34,35,36,37,38,39,40,41,42,43,44]. Therefore, the need for new ways of designing technology and the environment are needed, for instance, through the co-design and participatory approaches in research, while also considering and including the least-voiced in our societies, such as persons living with dementia or a physical limitation [37,45,46,47,48,49,50].

In this special issue, a rich pallet of views and studies has been presented. We acknowledge that this is only the tip of the iceberg and it contributes to (long-term) research regarding the interplay between people, the environment, and technology. We would like to call upon the wider scientific community, concerning both scholars engaged in fundamental or applied sciences, to use a wide array of research methodologies while using technological possibilities (including GIS, GPS, sensor technologies, advanced statistical techniques) combined with a rich set of qualitative data. This would enable research for exploring the interplay between human behavior, technology and the environment in its many shapes and facets. In this sense, the most important question is if and how this interplay contributes to the actual quality of life of people. This lies truly at the heart of the matter!
